# Supplementary material for: Resolving the full spectrum of human genome variation using Linked-Reads
Source: Genome Res. 2019 Apr;29(4):635–45. doi: 10.1101/gr.234443.118 (PMC6442396; doi:10.1101/gr.234443.118)
Supplement: Supplemental Material [file supp_gr.234443.118_Supplemental_longranger-2.2.zip › longranger-2.2.tar.bz2!/lr2.2-public/lib/stan/cmdstan/stan/doc/api/html/namespacemembers_func_m.html]

Stan: Namespace Members


|  |  |
| --- | --- |
|  | Stan  2.5.0  probability, sampling & optimization |


- Main Page
- Related Pages
- Namespaces
- Classes
- Files

- Namespace List
- Namespace Members

- All
- Functions
- Variables
- Typedefs
- Enumerations
- Enumerator

- a
- b
- c
- d
- e
- f
- g
- h
- i
- j
- l
- m
- n
- o
- p
- q
- r
- s
- t
- u
- v
- w


All Classes Namespaces Files Functions Variables Typedefs Enumerations Enumerator Friends Macros Pages

### - m -

- machine\_precision()
  : stan::math
- make\_nu()
  : stan::prob
- matrix\_normal\_prec\_log()
  : stan::prob
- max()
  : stan::math
- max\_size()
  : stan
- max\_size\_mvt()
  : stan
- mdivide\_left()
  : stan::agrad
  , stan::math
- mdivide\_left\_ldlt()
  : stan::agrad
  , stan::math
- mdivide\_left\_spd()
  : stan::agrad
  , stan::math
- mdivide\_left\_tri()
  : stan::agrad
  , stan::math
- mdivide\_left\_tri\_low()
  : stan::agrad
  , stan::math
- mdivide\_right()
  : stan::agrad
  , stan::math
- mdivide\_right\_ldlt()
  : stan::math
- mdivide\_right\_spd()
  : stan::math
- mdivide\_right\_tri()
  : stan::math
- mdivide\_right\_tri\_low()
  : stan::agrad
  , stan::math
- mean()
  : stan::math
- min()
  : stan::math
- minus()
  : stan::math
- modified\_bessel\_first\_kind()
  : stan::agrad
  , stan::math
- modified\_bessel\_second\_kind()
  : stan::agrad
  , stan::math
- modulus()
  : stan::math
- multi\_gp\_cholesky\_log()
  : stan::prob
- multi\_gp\_log()
  : stan::prob
- multi\_normal\_cholesky\_log()
  : stan::prob
- multi\_normal\_cholesky\_rng()
  : stan::prob
- multi\_normal\_log()
  : stan::prob
- multi\_normal\_prec\_log()
  : stan::prob
- multi\_normal\_rng()
  : stan::prob
- multi\_student\_t\_log()
  : stan::prob
- multi\_student\_t\_rng()
  : stan::prob
- multinomial\_log()
  : stan::prob
- multinomial\_rng()
  : stan::prob
- multiply()
  : stan::agrad
  , stan::math
- multiply\_log()
  : stan::agrad
  , stan::math
- multiply\_lower\_tri\_self\_transpose()
  : stan::agrad
  , stan::math


---

[ Stan Home Page ]

*© 2011–2014,
Stan Development Team.*
